# Preliminary study: cognitive behavioural therapy for insomnia in adolescents with anorexia nervosa

**DOI:** 10.1007/s40519-023-01634-4

**Published:** 2024-01-04

**Authors:** Léna Crevits, Catarina Silva, Flora Bat-Pitault

**Affiliations:** 1 Child and Adolescent Psychopathology Unit, Salvator University Hospital, Public Assistance-Marseille Hospitals, Aix-Marseille University, 249 Boulevard Sainte-Marguerite, 13009 Marseille, France; 2 Adult Psychopathology Unit, Valvert Hospital, 78 Boulevard des Libérateurs, 13011 Marseille, France; 3 Institute of Neuroscience Timone, CNRS, Aix-Marseille University, Marseille, France

**Keywords:** Anorexia nervosa, Insomnia, Adolescents, Cognitive and behavioural therapy for insomnia, CBT-I

## Abstract

**Purpose:**

Insomnia and anorexia nervosa (AN) are frequently comorbid, negatively affecting the evolution and the prognosis of AN. Within this framework, the management of sleep disorders appears as critical. The aim of this retrospective study is to assess, for the first time, the efficacy of cognitive and behavioural therapy for insomnia (CBT-I) on sleep disturbances in adolescents with AN. To do so, we investigated the impact of CBT-I on sleep disturbances and sleep-related outcomes, in BMI, AN symptoms, anxiety and depressive symptoms, emotionality and quality of life. These features were compared between two groups of patients with AN, one following CBT-I, and the other receiving the regular treatment at the psychiatric unit.

**Methods:**

Data collection occurred between January and May 2022. The study included 42 adolescents in-treatment at the Eating Disorders care specialised unit at Salvator Hospital in Marseille. They were randomly assigned to the CBT-I group (*N* = 31) or the control group (*N* = 11). Several clinical elements were assessed using sleep diaries and self-report questionnaires.

**Results:**

Participants undergoing CBT-I showed a significant improvement in sleep latency, total wake time and sleep efficacy, as well as in physical well-being. No significant effects were found regarding AN symptoms.

**Conclusion:**

These preliminary findings provide support for CBT-I effectiveness in adolescents with AN, as shown by significant improvements in several sleep parameters, as well as in physical well-being. These promising results, underline the relevance of this topic and its potential benefits for a more appropriate treatment for adolescents with AN.

*Level of evidence:* Level V, retrospective study.

## Introduction

Anorexia nervosa (AN) is a particularly serious disorder beginning in most cases during adolescence [1] and with one of the highest all-cause mortality and suicide rates among psychiatric disorders [2]. Adolescence is indeed a sensitive period for the development of other disorders, such as insomnia [3, 4]. It has been suggested that this may be due to the combination of sleep maturation processes [5] with environmental factors [3, 4, 6]. Indeed, insomnia is a common and chronic disorder amongst adolescents [7], with an estimated prevalence of 10% [7, 8]. Its significant consequences on physical and mental health, daytime functioning and quality of life constitute a major public health concern [3, 4, 6, 8]. Insomnia is also a frequent comorbid disorder as well as a risk factor for the onset, maintenance and relapse of other psychiatric conditions [3–6, 9] and an increasing risk for suicidal ideation and suicide attempts [3, 4, 10]. In particular, sleep disorders are thought to increase the emergence of eating disorders [11–13]. On the other hand, patients with eating disorders seem to have more sleep disturbances [12–14] and to present higher risks of developing sleep disorders [13, 15]. Over 50% of patients with AN have sleep disorders [16], as accessed by both subjective and objective measures [16–19].

The combination of these two disorders further deteriorates physical and mental health and significantly affects the course, the treatment response, and the prognosis of AN [13, 20]. This is particularly worrying concerning the increasing risks of developing comorbid depression or anxiety disorders [18, 21], and the significant decrease of the already severely impaired quality of life of patients with AN [22]. Indeed, several authors have suggested that improving sleep disturbances could have a significant positive impact on the efficacy of the overall care of AN [13, 17, 21], as well as on its course and prognosis [13, 20]. Additionally, its successful outcome would not only reduce symptoms related to comorbid anxiety or depression disorders, but also significantly ameliorate emotional disturbances and the quality of life [17, 21]. This suggests that targeting sleep disturbances in the context of treatment interventions for AN may be critical for its success [13, 17–21]. So far, cognitive and behavioural therapy for insomnia (CBT-I) has been considered the best candidate to do so [12].

CBT-I is an evidence-based psychotherapeutic approach aiming at improving sleep. It focuses on maladaptive behaviours, habits, and sleep-related misconceptions and cognitions [23]. CBT-I has been shown to be effective, improving sleep parameters in adolescents, at both short and long term [6, 24, 25], with a beneficial impact on both psychiatric (e.g. anxiety and depression) and somatic comorbid pathologies, as well as in daytime functioning and quality of life [6, 26]. CBT-I has also shown to have a superior long-term effectiveness compared to benzodiazepine and hypnotic drugs, which are not recommend for sleep disorders in children and adolescents [4, 27]. Besides CBT-I does not induce the numerous adverse effects of these treatments [23, 28]. This approach is thus the first-line treatment recommended in France and internationally for the management of insomnia in adults [23, 28] and adolescents [29]. Moreover, patients with AN are more likely to manifest those side effects due to malnutrition, which also leads to metabolic disturbances and somatic complications that can interfere with medication intake [30, 31].

Within this framework, the aim of this retrospective study is to assess, for the first time (although previously suggested by several authors, see 12, 13, 17–21), the efficacy of CBT-I on sleep disturbances in adolescents with AN. By using a control group (not undergoing CBT-I) we investigated the impact of CBT-I on sleep disturbances and sleep-related outcomes, in BMI, AN symptoms, anxiety and depressive symptoms, emotionality and quality of life.

## Methods

### Participants

Forty-two adolescents undergoing a treatment at the Eating Disorders care specialised unit at Salvator Hospital in Marseille were included. All patients were diagnosed with AN by a specialised psychiatrist, according to DSM-5 criteria. Patients were assigned to one of two groups (CBT-I group, *N* = 31; and control group, *N* = 11) depending on their admission date at the hospital unit. Nine patients were excluded due to unsatisfactory data collection (incomplete questionnaires, no sleep diary returned). The study thus included data from 33 patients: 22 patients were included in the CBT-I group and 11 patients included in the control group.

### Procedures

Data collection occurred between January and May 2022. Participants included were either in partial hospitalisation (day hospitalisation) or full hospitalisation (inpatients). The patients were not taking any z-drugs neither benzodiazepines but 5 of them (2 in the control group and 3 in the CBT-I group) were taking melatonin since at least 4 weeks, and without any dose changes. Those in the control group followed a classic intervention program for AN. This involves re-nutrition and therapeutic groups for the patient and the family. The CBT-I program used included the classic and validated axes of behavioural techniques (sleep restriction therapy and stimulus control), cognitive therapy, and sleep-related psychoeducation. Previous studies suggest that group therapy is efficient [32] and that 4 sessions is the optimal dosing for individual therapy [33]. Following these guidelines, the program was delivered by a psychiatrist in 4 sessions of 1 h each over of a 5-week period and included a maximum of 8 patients per group. All patients, both included in the CBT-I group and in the control group were assessed before (T0) and after therapy (T1, that is, after 5 weeks). The assessment common to all patients included clinical data (age, weight and height for BMI calculation) and self-report questionnaires. Patients also filled in a sleep diary daily between T0 and T1, from which several variables were determined: total sleep time (TST), time spent in bed, wake after sleep onset (WASO), sleep latency, total wake time (from lights out to wake up time) and sleep efficiency (calculated as "total sleep time/time in bed" × 100).

### Materials

Sleep parameters were assessed using several measures: (1) the ESS-CHAD (Epworth Sleepiness Scale for Children and Adolescents), an 8-item scale used for assessing daytime sleepiness. Scores greater than 10 suggest pathological daytime sleepiness [34]; (2) the Insomnia Severity Index (ISI), a 7-item scale used to assess insomnia severity for which the total score ranges from 0 to 28 (< 7 = normal sleep; 8–14 = mild insomnia; 15–21 = moderate insomnia; > 22 = severe insomnia) [35]; and (3) the Pittsburgh Sleep Quality Index (PSQI). The PSQI assesses sleep quality and disturbance over a 1-month period. It is composed of 19 items providing an overall score ranging from 0 to 21: the higher the score, the greater the sleep disturbances. Scores over 5 indicate poor sleep quality [36].

Eating symptomatology was measured using the Eating Disorder Inventory 2 (EDI-2) [37]. The EDI-2 is composed of 91 items tapping eating attitudes and behaviours related to AN and bulimia nervosa. Items are rated on a 6-point Likert scale (from 0 = never, to 5 = always). The higher the total score (the sum of all dimensions), the greater the eating psychopathology.

Depressive elements were assessed with the Children's Depression Inventory (CDI) [38, 39]. The CDI is composed of 27 items. The total (sum) score ranges from 0 to 54. The threshold for depression is 15.

Anxiety was assessed using the STAI-Y (State Trait Anxiety Inventory) [40, 41]. The STAY-Y assesses anxiety *at the present moment* (form STAI-Y-1, 20 items) and in *general* (form STAI-Y-2, 20 items). For both scales, scores range from 20 to 80: scores of 35 or less = very low anxiety; scores > 65 = very high anxiety.

Quality of Life (QoL), was measured using the "Vécu et Santé Perçue de l'Adolescent" (VSP-A) scale [41]. The VSP-A comprises 37 items divided into 10 dimension including psychological and physical well-being, and an overall quality-of-life index. Greater scores indicate higher QoL [42].

The Positive and Negative Emotionality Scale (EPN-31) [43] assessed emotionality with 31 items, considering the frequency of 31 basic or more elaborated emotions experienced over the past month. Three main scores are calculated for positive, negative and surprise affects.

### Statistical analysis

Statistical analyses were carried out using the SPSS 20.0 software. Due to the small sample size, between groups comparisons were analysed using non-parametric Mann–Whitney U tests. We first compared clinical features of both groups at the initial, pre-therapy assessment, T0. We then used Wilcoxon test to compare those features before (T0) and after CBT-I (T1). All statistical tests were performed bilaterally with an alpha level of 5% and *p* < 0.05 were considered significant.

## Results

### Before therapy: T0

The socio-demographic and clinical features, as well as the responses to the self-report questionnaires and sleep diary of both groups at the beginning of the program (T0) are presented in Table 1. Because between-group analyses revealed no significant differences between the two groups at T0, we therefore pursue by conducting within-group analyses. This allowed exploring the evolution within each group. Analyses showed that (see Table 1) both groups did not differ in the majority of the variables assessed. Overall, adolescents in both groups reported mild insomnia rates (ISI), moderate sleep disturbances (PSQI), presence of anxiety (STAY) and depressive (CDI) symptoms, as well as a severely impaired quality of life and a greater negative emotionality. The two groups differed only in terms of anxiety. The CBT-I group reported significantly higher anxiety than the control group, although scores in both groups suggests moderate anxiety.

**Table 1:** Clinical features (means and standard deviations) of adolescents with AN in the control and the CBT-I groups at T0 ( Mann–Whitney U tests for between groups comparisons)

	Control Group (*N* = 11)		CBT-I Group (*N* = 22)		*p*
	Mean	SD	Mean	SD	
Age (years)	15.65	1.52	15.41	1.20	ns
BMI (kg/m^2^)	15.34	1.55	16.34	1.86	ns
ESS	7.86	7.37	11.52	5.61	ns
ISI	9.14	8.51	9.95	5.77	ns
PSQI total score	7.82	3.94	8.48	4.30	ns
EDI-2 total score	93.00	79.54	119.14	55.76	ns
CDI total score	19.00	9.77	23.45	9.41	ns
STAI Form Y1	**48.91**	**15.45**	**61.32**	**13.19**	**0.011**
VSPA					
Psychological wellness	30.45	21.96	23.33	19.26	ns
Physical wellness	58.45	25.42	46.52	22.36	ns
QoL total score	41.36	14.41	35.52	12.53	ns
EPN-31					
Positive emotions	47.00	12.88	41.20	13.23	ns
Negative emotions	78.20	29.35	97.10	21.84	ns
Sleep diary					
Bedtime (h)	22.36	0.54	22.34	0.63	ns
Sleep latency (min)	27.64	15.89	49.83	51.23	ns
Wake time (h)	7.51	0.93	7.81	0.91	ns
Total sleep time (h)	8.00	1.00	7.29	1.29	ns
Time in bed (h)	8.75	0.67	9.46	0.86	ns
WASO (min)	17.37	20.32	46.10	35.56	ns
Total wake time (h)	0.77	0.67	1.59	1.08	ns
Sleep efficacy (%)	91.42	40.27	77.52	13.97	ns

Significant differences are indicated in bold

Regarding the parameters assessed by the sleep diary (see Table 1), analyses also showed no significant differences between the groups. This overall suggests that patients included in the two groups were homogeneous and comparable.

### After therapy: T1

Table 2 presents the evolution of sleep, eating and psychiatric symptomatology between T0 and T1 for all patients in the two groups. Analyses showed that for patients that did not follow CBT-I, body weight (BMI) was the only variable that changed significantly between T0 and T1. Patients gained weight during the typical in-treatment procedures at the unit. This was also the case of the patients on the CBT-I group. Importantly, results showed that after CBT-I patients showed a significant reduction in sleep latency and in total wake time, as well as a significant amelioration of sleep efficacy. Furthermore, there was also a significant improvement of physical wellness in these patients.

**Table 2:** Clinical and sleep features (means and standard deviations) of adolescents with AN before (T0) and after (T1) the therapy in the control and the CBT-I groups (Wilcoxon tests for within groups comparisons)

	Control group (N = 11)				*p*	CBT-I group (N = 22)				*p*
	T0		T1			T0		T1		
	Mean	SD	Mean	SD		Mean	SD	Mean	SD	
BMI (kg, m^2^)	**15.34**	**1.55**	**15.89**	**1.41**	**0.01**	**16.34**	**1.86**	**16.90**	**1.86**	**0.001**
ESS	7.86	7.37	8.86	6.15	ns	11.52	5.61	10.33	6.04	ns
ISI	9.14	8.15	9.29	7.72	ns	9.95	5.77	9.24	6.12	ns
PSQI total score	7.82	3.94	7.27	3.93	ns	8.48	4.30	7.52	5.05	ns
Sleep diary										
Bedtime (h)	22.36	0.54	22.56	22.56	ns	22.34	0.63	22.66	0.75	ns
Sleep latency (min)	27.64	15.89	21.36	21.36	ns	**49.83**	**51.23**	**34.13**	**40.55**	**0.02**
Wake time (h)	7.51	0.93	8.14	8.14	ns	7.81	0.91	7.96	0.99	ns
Total sleep time (h)	8.00	1.00	8.23	8.23	ns	7.29	1.29	7.48	1.44	ns
Time in bed (h)	8.75	0.67	9.18	9.18	ns	9.46	0.86	9.29	1.02	ns
WASO (min)	17.37	20.32	21.64	21.64	ns	46.10	35.56	43.83	41.84	ns
Total wake time (h)	0.77	0.67	0.92	0.92	ns	**1.59**	**1.08**	**1.30**	**1.09**	**0.04**
Sleep efficacy (%)	91.42	40.27	89.65	89.65	ns	**77.52**	**13.97**	**81.03**	**14.04**	**0.01**
EDI-2 total score	93.00	79.54	94.60	94.60	ns	119.14	55.76	119.36	53.38	ns
CDI total score	19.00	9.77	18.91	18.91	ns	23.45	9.41	24.59	10.82	ns
STAI Form Y-1	48.91	15.45	49.91	49.9	ns	61.32	13.19	62.55	14.34	ns
VSPA										
Psychological wellness	30.45	21.96	33.64	33.64	ns	23.33	19.26	22.86	20.41	ns
Physical wellness	58.45	25.42	55.64	55.64	ns	**46.52**	**22.36**	**54.90**	**17.70**	**0.01**
QoL total score	41.36	14.41	44.45	44.45	ns	35.52	12.53	35.10	14.30	ns
EPN-31										
Positive emotions	47.00	12.88	45.20	45.20	ns	41.20	13.23	41.00	13.11	ns
Negative emotions	78.20	29.35	81.00	81.00	ns	97.10	21.84	94.90	24.81	ns

Significant differences are indicated in bold

## Discussion

Although suggested by several authors, this is, to our knowledge, the first study investigating the potential efficacy of CBT-I as a sleep therapy in AN. In agreement with the literature, our results showed significant sleep disturbances in patients with AN. According to the sleep diary, patients reported a total sleep time of about 7 to 8 h before the therapy, which is below the recommended sleep duration of 8 to 10 h for the age group of our patients [44]. These findings are consistent with others reporting a frequently decreased in total sleep time in patients with AN [17]. In line with this and consistent with others [16, 18] the patients included here reported the presence of moderate sleep disorders and of mild insomnia rates (as measured by the PSQI and ISI scores, respectively). Overall present findings showed CBT-I effectiveness targeting sleep disturbances in AN. In particular, CBT-I revealed significant improvements in several sleep parameters such as sleep latency, sleep efficiency and total wake time. These post-therapy improvements were already described in the literature with other populations [6, 24, 25]. Our study contributes to the field showing the same effects in an adolescent sample with AN. On the other hand, no significant differences were found at the end of therapy regarding total sleep time, time spent in bed and WASO. However, studies investigating CBT-I effectiveness in these sleep parameters have shown inconsistent results (e.g. 6, 24, 25). In this specific sample of patients with AN, patients’ perception is often thought to be altered due to a body–mind dissociation experienced, which has been previously described [45], as well as a poor insight [46]. This can explain an advantage of the patients’ perceptions assessed by the objective measures (sleep diary), prior to subjective measures (ISI) in the beginning of the therapy.

Our results also show a diminished quality of life in these patients. Other studies found this same tendency, both in patients with AN [22] and with insomnia [4, 8]. At the end of the therapy, a significant improvement in physical well-being was found in the CBT-I group. This positive impact of CBT-I in quality of life and physical well-being has also been previously reported [47]. This is a promising finding, as physical well-being is known to be strongly affected in AN [22], and suggests that CBT-I may be an adapted strategy to improve it. The stability of the overall quality of life score before and after therapy was less surprising. Studies indicate that CBT-I may be less effective improving quality of life when another pathology is comorbid to insomnia [47], which is the case here. Along with a diminished quality of life, patients also seem to experience negative emotions more often. This result has been frequently found in adolescents with insomnia [10, 24]. In the present study, no significant changes were found in the experienced emotionality at the end of the therapy. No significant differences were found in overall symptoms of AN either. This was perhaps due to the small sample size on one hand, and to the lack of a long-term follow-up on the other hand, which would have enabled us to observe a potential evolution.

Assessment of psychiatric symptoms showed comorbid anxiety and depressive symptoms (as measured by the STAI-Y and the CDI, respectively), which has been widely described in the AN literature (e.g. [16, 48]). In addition, comorbid insomnia also increases the risk of developing these symptoms [18, 21]. Nonetheless, no significant amelioration in anxiety and depressive symptoms after CBT-I were found in our sample of adolescents, despite other studies showing otherwise [26]. The lack of CBT-I effectiveness in these parameters may be explained by the fact that AN favours the maintenance of anxiety and depressive symptoms by itself. This underlines the importance of targeting simultaneous interventions in AN, sleep disorders, and anxiety and depressive symptoms, which can persist after an initial phase of re-nutrition. Indeed, it would have been interesting to evaluate the long-term effects of CBT-I in these symptoms as well as the improvements found in sleep parameters, as studies suggests a progressive evolution of the sleep parameters and anxiety and depressive symptoms over time [6, 33]. Analyses also showed a significant increase of the BMI at T1 in both groups. Improvements in sleep and physical well-being could also be related to weight gain and the consequent advantages of the nutritional status. However, it has been shown that nutritional status alone cannot explain sleep disturbances observed in AN and that weight gain is not sufficient to improve them [17, 49].

Despite its valuable contribution, this study has some limitations. Primarily we should mention its retrospective nature and the obvious constraints of the small sample size. The subjective report of sleep disturbances without objective measurement methods (e.g. actigraphy) is also a limitation. Indeed, patients with insomnia may tend to overestimate their sleep disturbances [50] and adolescents in particular, may have an inaccurate perception of sleep parameters changes or improvements [6]. Although much of the research on sleep are conducted using the participants subjective responses, [23, 28], future studies should consider objective measurement methods. This would undeniably provide consistency to the findings, and thereby a deeper comprehension of their real sleep disturbances.

To conclude, this preliminary study provides support for CBT-I effectiveness when targeting sleep disturbances. Here we show that this intervention also proves its efficacy among patients with AN. In particular, present findings show that CBT-I improves several sleep parameters, as well as physical well-being. These are promising results, which we believe, are worth further investigating. Different adaptations of CBT-I to the specific needs of adolescents with AN could be explored. In particular, greater parental involvement, as it has been suggested to play a key role in the success of the treatment in both children [4, 10] and adolescents [51], or coupling CBT-I with mindfulness meditation, targeting WASO improvements [52] which appear to be particularly increased in patients with AN [17, 19]. In this study we chose to include adolescents with AN regardless of their degree of sleep complaints. This seemed appropriate, considering the difficulty these patients have to report their sleep disturbances, and the importance of improving their sleep for the overall treatment. This must have had an impact on their motivation to undergo therapy, and therefore, on CBT-I effectiveness.

## Data Availability

The datasets generated and analyzed during the current study are available from the corresponding author on reasonable request.
